# Oblique lumbar interbody fusion combined with anterolateral screw fixation for treating severe lumbar spinal stenosis: Case series

**DOI:** 10.1097/MD.0000000000046658

**Published:** 2025-12-12

**Authors:** Xingrui Peng, Tianhang Xie, Xiandi Wang, Long Zhao, Xingxiao Pu, Xiao Hu, Xu Han, Liyu Ran, Jiancheng Zeng

**Affiliations:** a Department of Orthopedic Surgery and Orthopedic Research Institute, West China Hospital, Sichuan University, Chengdu, Sichuan, China.

**Keywords:** Oblique lumbar interbody fusion, minimally invasive, severe lumbar spinal stenosis, indirect decompression, anterolateral screw fixation

## Abstract

It is controversial for indirect decompression like oblique lumbar interbody fusion (OLIF) for treating severe lumbar spinal stenosis (SLSS). OLIF combined with anterolateral screw fixation (AF) can achieve decompression and fixation by single incision, which is minimally invasive. In this study, we aimed to evaluate the effectiveness and safety of OLIF–AF for treating SLSS. A retrospective study which comprised patients diagnosed with L4–5 SLSS of Schizas grade C or D caused by degenerative conditions who underwent L4–5 OLIF–AF with a follow-up of 24 months. Preoperative Visual Analog Scale score of low-back pain and leg pain, and Oswestry Disability Index, disc height, foraminal area, ligamentum flavum thickness, difference of left and right facet angle, and cross-sectional area (CSA) were compared to their 1 day, 3 months, 12 months, and 24 months postoperative counterparts. Eighty-two patients of L4–5 SLSS underwent OLIF–AF were included in this retrospective study. Ligamentum flavum thickness thinned from 4.94 ± 1.20 mm to 4.20 ± 0.95 mm after the operation, and 3.91 ± 0.78 mm and 3.87 ± 0.75 mm (all *P* < .05) at 12 and 24 months postoperatively. CSA expanded from 52.44 ± 15.38 mm^2^ to 103.98 ± 20.44 mm^2^ on postoperative day 1, and expand to 115.77 ± 20.04 mm^2^ and 117.71 ± 20.83 mm^2^ (all *P* < .05) at each annual follow-up. Visual Analog Scale score of low-back pain improved from 6.40 ± 0.99 to 2.56 ± 0.85 at 3 months postoperatively, then reached 1.55 ± 0.69 and 1.12 ± 0.64 (all *P* < .05) at each annual follow-up. Visual Analog Scale score of leg pain gradually improved from 6.16 ± 1.25 to 2.37 ± 0.82, 1.36 ± 0.71, and 1.10 ± 0.69 (all *P* < .05) at each time point. Oswestry Disability Index increased from 37.54 ± 6.01 to 18.81 ± 4.38 (*P* < .05) after surgery. And recovered to 12.42 ± 2.44 (*P* < .05) and 10.58 ± 1.63 (*P* < .05) at 1 and 2 years postoperatively. OLIF–AF can effectively restore disc height, foraminal area, and CSA, reducing lumbar rotation, thinning the hypertrophic ligamentum flavum, significantly improving patients’ pain and function, and maintaining efficacy, indicating that OLIF–AF may be a safe and effective treatment for SLSS.

## 1. Introduction

Lumbar spinal stenosis (LSS) is a common condition, with over 30% cases classified as severe lumbar spinal stenosis (SLSS).^[1]^ It is characterized by symptoms such as low-back pain, radiating leg pain, numbness, and neurogenic claudication, significantly impacting the quality of life for affected individuals.^[2]^

There is no consensus on the treatment of LSS, but typically conservative treatments are attempted first.^[3]^ If conservative treatments fail, surgery is necessary.^[4]^ The most common surgical approaches are traditional posterior surgeries like transforaminal lumbar interbody fusion (TLIF).^[5]^ However, with the advancement of society and medical technology, patients have increasingly emphasized reducing surgical trauma and shortening hospital stays. In recent years, minimally invasive spine surgeries have rapidly developed, leading to the emergence of procedures such as direct lateral interbody fusion, extreme lateral interbody fusion (XLIF), anterior lumbar interbody fusion (ALIF), midline lumbar interbody fusion, minimally invasive TLIF, lateral lumbar interbody fusion (LLIF), oblique lumbar interbody fusion (OLIF), and crenel lateral interbody fusion.^[6–8]^ Nevertheless, posterior surgeries all involve varying degrees of damage to posterior structures such as bones, muscles, and ligaments.^[9]^ Direct lateral interbody fusion, XLIF, LLIF, and CLIF require intraoperative neurophysiological monitoring, and ALIF carries the risk of damaging the abdominal organs or major vessels during the exposure process, making OLIF indirect decompression more advantageous.^[6,7,9]^

There is limited research on surgical treatment for SLSS, particularly regarding OLIF. Previous studies demonstrated OLIF combined with pedicle screw fixation (PSF) effectively treated SLSS and showed significant advantages over TLIF and PLIF.^[10,11]^ However, PSF requires change in positioning during surgery and extra incisions, which can prolong surgical time and may increase risk of infection and anesthesia-related complications. We have utilized the OLIF combined with anterolateral screw fixation (AF) technique, which allows for the completion of all procedures through a single incision.^[12]^ This technique has successfully treated various degenerative lumbar spine conditions and achieved good therapeutic outcomes.^[12]^ However, there is controversy regarding its use in treating SLSS, especially on whether it can achieve satisfying decompression. We hypothesized OLIF–AF safe and effective in treating SLSS. Therefore, we stared this study.

## 2. Methods

This retrospective study was performed in line with the principles of the Declaration of Helsinki. Approval was granted by the Ethics Committee of West China Hospital of Sichuan University (approval number: 2023-2285). Informed consent was obtained from all individual participants included in the study.

Inclusion criteria: patients diagnosed with L4–5 LSS of Schizas grade C or D; stenosis caused by degenerative lumbar spondylolisthesis of Meyerding grade I or II, or degenerative lumbar spinal stenosis with instability; patients who have undergone conservative treatment for at least 3 months without relief of low-back pain or leg pain; dual-energy X-ray absorptiometry *T* value > -2.5 SD; minimum follow-up of 24 months. Lumbar instability was defined as Wood et al described.^[13]^

Exclusion criteria: patients with intraoperative endplate injury; previous lumbar surgery history; patients with spinal tumors or infectious diseases; Bony stenosis of lumbar spinal canal.

### 2.1. Surgical methods

Following induction of general anesthesia and intubation, patients were positioned in lateral decubitus posture. Using C-arm fluoroscopy, target intervertebral disc was located. A 5-cm incision was made in lateral abdominal region, parallel to iliac crest. Employing a muscle-splitting technique, the external oblique, internal oblique, and transverse abdominal muscles were dissected along their fibers. Blunt dissection was utilized to access the retroperitoneal space, with the peritoneal contents mobilized anteriorly. The psoas muscle was identified and split at its anterior one-third. This section of the psoas muscle was then retracted ventrally to expose the surgical area while protecting ureter and major blood vessels. Once the L4–5 intervertebral disc and the lateral side of the adjacent vertebral body were visualized, C-arm fluoroscopy confirmed proper positioning before proceeding with interbody fusion. A tubular retractor system was attached. After the cage-posterior longitudinal ligament area decompression technique, then derotation and vertebral spondylolisthesis reduction were accomplished by trail fitting, and the vertebral endplates were prepared for cage insertion. A cage filled with synthetic bone containing bone morphogenetic protein-2 was inserted. The spinal canal was reshaped and broadened. Screws were placed on lateral side of vertebrae, near the endplate. A connecting rod was then installed and locked. Finally, the abdominal muscle planes and skin were closed in layers.

**Figure 1.**
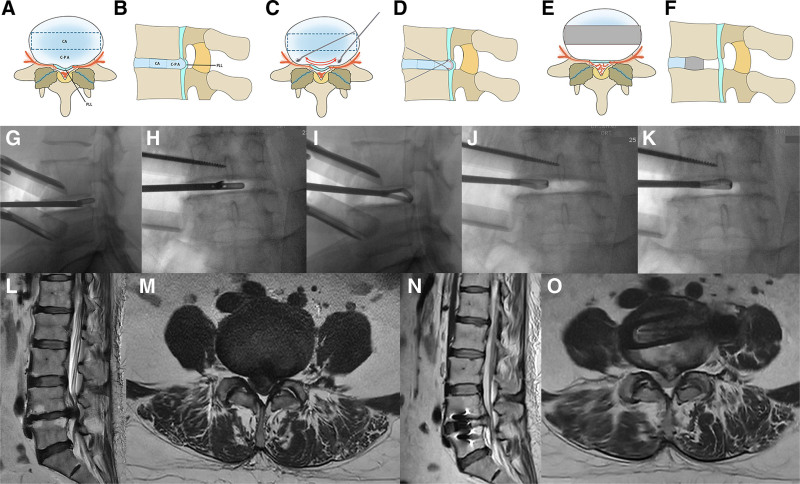
Procedure of C-P A decompression. (A, B) Definition of CA and C-P A, and C-P A is the area from cage to posterior longitudinal ligament, which includes backward disc. (C, D) After removing disc of CA and C-P A, thin and release PLL with curette, especially near the upper and lower endplates. (E, F) After C-P A decompression and cage insertion, PLL was thinned and restored, ligamentum flavum was thinned, DH, FA, and CSA were increased. (G–K) Intraoperative images of removing disc and thinning PLL. (J, M) Sagittal and axial plane of preoperative MRI of a typical patient of L4–5 SLSS. (N, O) Sagittal and axial plane of postoperative MRI of the same patient. CA = cage area; C-P A = cage-posterior longitudinal ligament area, CSA = cross-sectional area, DH = disc height, FA = foramen area, MRI = magnetic resonance imaging, PLL = posterior longitudinal ligament, SLSS = severe lumbar spinal stenosis.

Cage-posterior longitudinal ligament area (C-P A) decompression: we defined the area from cage to posterior longitudinal ligament as C-P A, which includes backward disc. Firstly, we completely remove the disc at the position which planned for cage placement, and the disc space at the C-P A. Then thin and release posterior longitudinal ligament with curette, especially near the upper and lower endplates. The whole C-P A decompression procedure was completed (Fig. 1).

### 2.2. Radiological measurements

Preoperative, 1-day, 3-month, 12-month, and 24-month postoperative lumbar X-ray and 3-dimensional computed tomography (3D-CT) scans were collected, along with preoperative, 1-day, 12-month, and 24-month postoperative magnetic resonance imaging (MRI) scans. Foramen area (FA) was measured on lateral X-ray. On the 3D-CT scans, measurements were taken for disc height (DH), difference of left and right facet angle (DFA). On MRI scans, ligamentum flavum thickness (LF) and cross-sectional area (CSA) were measured. DH and CSA were measured as former research.^[14]^ FA was measured as Park et al conducted, LF was measured as Takahashi et al conducted.^[15]^ DFA was defined as the absolute value between left and right facet angle. Cage subsidence (CS) was defined as change in DH >2 mm after the operation, and if it reached 4 mm, it was defined as severe (change in DH = DH at 24 months postoperatively - DH at 1 day postoperatively). CS was classified as mild if change in DH ranged from 2 to 4 mm and severe if it exceeded 4 mm. Prior to the operation, bone mineral density (BMD) was assessed using dual-energy X-ray absorptiometry, specifically recording the minimum *T* value of the hip. Fusion status was evaluated using 3D-CT scans, and the fusion grade criteria followed as Bridwell et al described.^[16]^ Grade 1 and 2 indicated fusion. Two authors independently performed measurements of the remaining continuous variables, and all were averaged. All radiological measurements were conducted on picture archiving and communication system.

### 2.3. Clinical evaluation

All participants were followed up at least 24 months. Sex, age, body mass index, BMD, operation duration, estimated blood loss, and hospital stay, were recorded. Preoperatively, as well as at 3, 12, and 24 months postoperatively, Visual Analog Scale (VAS) scores for low-back pain (VAS-LBP) and leg pain (VAS-LP), and Oswestry Disability Index (ODI) were documented. Follow-up was conducted through clinic visits or telephone interviews. Any surgical-related complications were thoroughly documented.

### 2.4. Statistical analysis

Data analysis was performed using GraphPad Prism 10.1.2 (GraphPad Software, CA, USA). Numerical continuous variables were presented as mean ± standard deviation. Changes in DH, FA, LF, DFA, CSA, VAS-LBP, VAS-LP, and ODI scores were evaluated using repeated-measures ANOVA with Tukey multiple comparisons test. *P* < .05 was considered statistically significant.

## 3. Results

### 3.1. General data

This study comprised 82 patients (22 males and 60 females) who diagnosed L4–5 SLSS underwent L4–L5 OLIF–AF between March 2018 and December 2022. Their age was 68.1 ± 10.8 years, BMD and body mass index were -1.8 ± 0.3 SD and 22.5 ± 1.5 kg/m^2^. Operation duration, estimated blood loss, and hospitalization were 61.1 ± 9.2 minutes, 20.9 ± 5.7 mL, 5.2 ± 1.0 days, respectively (Table 1).

**Table 1:** General data.

Sex (male:female)	22:60
Age (yr)	68.1 ± 10.8
BMI (kg/m^2^)	22.5 ± 1.5
BMD (*T* value SD)	-1.8 ± 0.3
Operation duration (min)	61.1 ± 9.2
Estimated blood loss (mL)	20.9 ± 5.7
Hospitalization (d)	5.2 ± 1.0

Data presented as mean ± standard deviation.

BMD = bone mineral density, BMI = body mass index; n = number of patients.

### 3.2. Radiological evaluation

**Figure 2.**
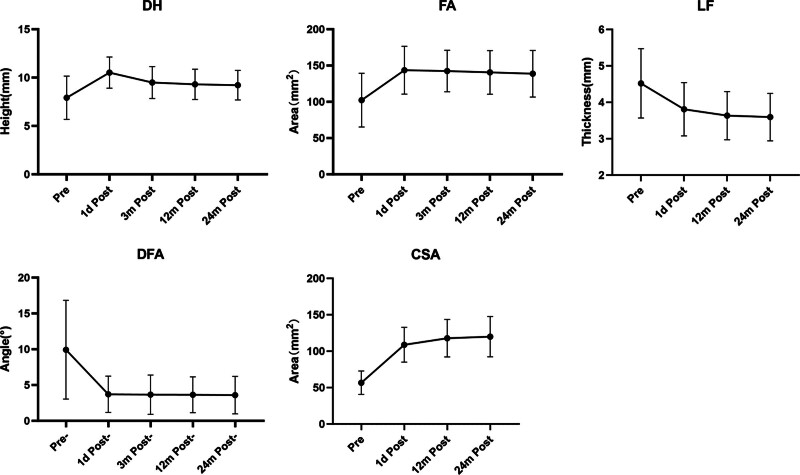
Radiographic data at different time points of the follow-up. CSA = cross-sectional area; DFA = absolute value of difference between left and right facet angle; DH = disc height; FA = foramen area; LF = ligamentum flavum thickness.

DH increased from preoperative 7.88 ± 1.79 to 10.46 ± 1.35 mm 1 day postoperatively, and decreased to 9.42 ± 1.41, 9.24 ± 1.37, 9.17 ± 1.36 mm every next follow-up (all *P* < .05). Similarly, FA increased from preoperative 96.22 ± 29.57 mm^2^ to 136.12 ± 29.52 mm^2^ after the operation, and then gradually decreased to 134.14 ± 26.05, 132.36 ± 26.80, and 130.73 ± 27.52 mm^2^ at 3, 12, and 24 months postoperatively, respectively (all *P* < .05). DFA reduced from 11.74 ± 7.25° to 4.03 ± 2.78° 1 day postoperatively, 4.02 ± 2.85° 3 months postoperatively, 4.02 ± 2.76° and 4.01 ± 2.82° at postoperative 1 and 2 years respectively (all *P* < .05). LF thinned from 4.94 ± 1.20 to 4.20 ± 0.95 mm after the operation, and 3.91 ± 0.78 mm at 12 months postoperatively, 3.87 ± 0.75 mm 24 months postoperatively (all *P* < .05). CSA expanded from preoperative 52.44 ± 15.38 to 103.98 ± 20.44 mm^2^ on postoperative day 1, and continued to expand to 115.77 ± 20.04 mm^2^ at 1 year postoperatively (all *P* < .05). Then reached 117.71 ± 20.83 mm^2^ at 2 year postoperatively (*P* < .05). Spinal stenosis ranking, CS, and fusion status were all recorded. CS occurred in 14 cases but all were mild, and fusion was achieved in all cases in 2 years (Tables 2 and 3, Fig. 2).

**Table 2:** Radiographic data.

	Pre-	1 d post-	3 mo post-	12 mo post-	24 mo post-
DH (mm)	7.88 ± 1.80	10.46 ± 1.35*	9.42 ± 1.41*†	9.24 ± 1.37*†	9.17 ± 1.36*†
FA (mm^2^)	96.22 ± 29.57	136.12 ± 29.52*	134.14 ± 26.05*	132.36 ± 26.80*	130.73 ± 27.52*
LF (mm)	4.94 ± 1.20	4.20 ± 0.95*	–	3.91 ± 0.78*	3.87 ± 0.75*
DFA (°)	11.74 ± 7.25	4.03 ± 2.78*	4.02 ± 2.85*	4.02 ± 2.76*	4.01 ± 2.82*
CSA (mm^2^)	52.44 ± 15.38	103.98 ± 20.44*	–	115.77 ± 20.04*†	117.71 ± 20.83*†
Spinal stenosis status		Pre-	1 d Post-	12 m Post-	24 m Post-
Schizas grade A		0	25	32	32
Schizas grade B		0	19	50	50
Schizas grade C		45	28	0	0
Schizas grade D		37	0	0	0

Data presented as mean ± standard deviation.

CSA = cross-sectional area, DFA = absolute value of difference between left and right facet angle, DH = disc height, FA = foramen area, LF = ligamentum flavum thickness, ODI = Oswestry Disability Index, VAS-LBP = Visual Analog Scale of low-back pain, VAS-LP = Visual Analog Scale of leg pain.

* *P* < .05 compared to pre-.

† *P* < .05 compared to 1 d post-.

**Table 3:** CS and fusion status.

	Yes	No
CS (n)	14 (17.07%)	68 (82.93%)
Fusion (n)		
12 m Post-	74 (90.24%)	8 (9.76%)
24 m Post-	82 (100%)	0

CS = cage subsidence, n = number of patients, post- = postoperative.

### 3.3. Clinical and functional evaluation

**Figure 3.**
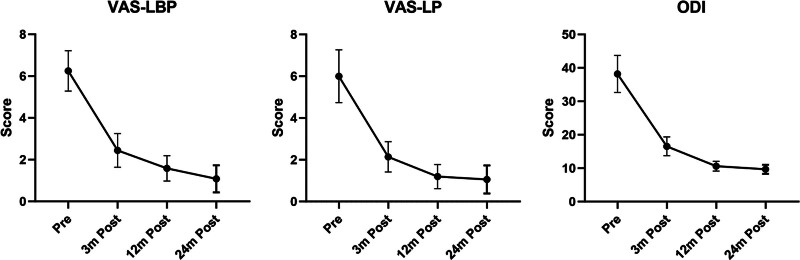
Pain and functional data. ODI = Oswestry Disability Index; VAS-LBP = Visual Analog Scale score of the low-back pain; VAS-LP = Visual Analog Scale score of leg pain.

VAS-LBP decreased from preoperative 6.40 ± 0.99 to 2.56 ± 0.85 at 3 months postoperatively, then reached 1.55 ± 0.69 and 1.12 ± 0.64 at 1 and 2 years postoperatively (all *P* < .05). VAS-LP improved from 6.16 ± 1.25 to 2.37 ± 0.82, 1.36 ± 0.71 and 1.10 ± 0.69 at each follow-up (all *P* < .05). ODI improved from 37.54 ± 6.01 to 18.81 ± 4.38 after surgery (*P* < .05). And recovered to 12.42 ± 2.44 and 10.58 ± 1.63 each annual follow-up (all *P* < .05) (Table 4, Fig. 3).

**Table 4:** Pain and functional data.

	Pre-	3 m Post-	12 m Post-	24 m Post-
VAS-LBP	6.40 ± 0.99	2.56 ± 0.85*	1.55 ± 0.69*†	1.12 ± 0.64*†‡
VAS-LP	6.16 ± 1.25	2.37 ± 0.82*	1.36 ± 0.71*†	1.10 ± 0.69*†
ODI	37.54 ± 6.01	18.81 ± 4.38*	12.42 ± 2.44*†	10.58 ± 1.63*†‡

Data presented as mean ± standard deviation.

ODI = Oswestry Disability Index, VAS-LBP = Visual Analog Scale of low-back pain, VAS-LP = Visual Analog Scale of leg pain.

* *P* < .05 compared to pre-.

† *P* < .05 compared to 3 m post-.

‡ *P* < .05 compared to 12 m post-.

### 3.4. Complications

**Figure 4.**
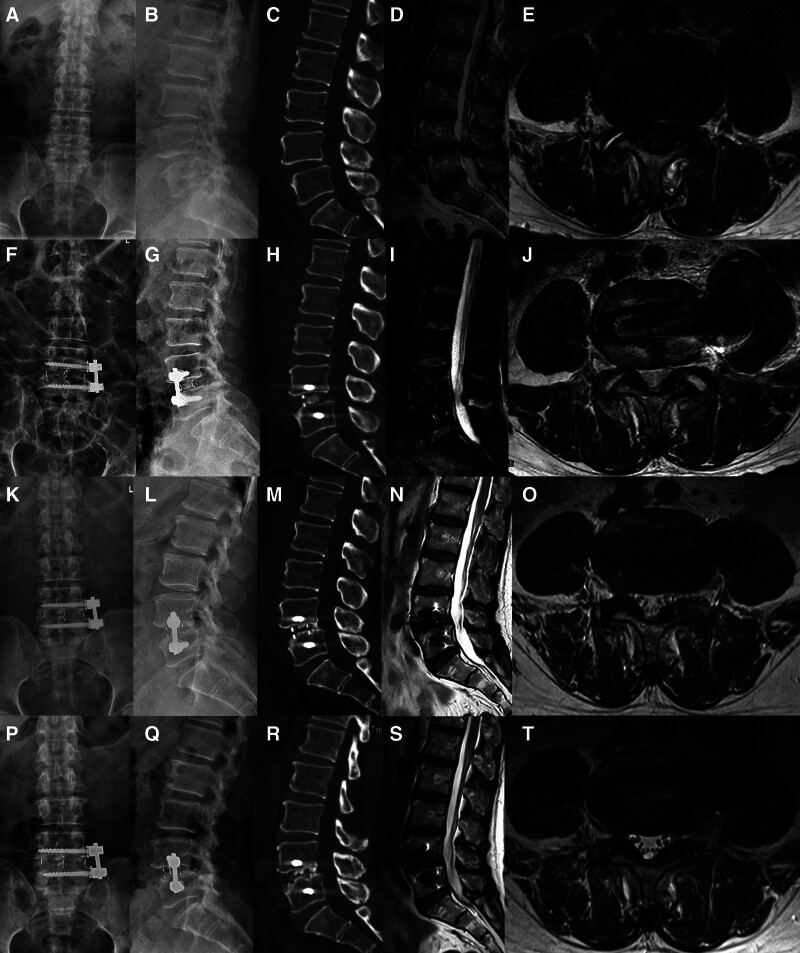
A patient of L4–L5 SLSS underwent OLIF–AF. (A–E) A–P and lateral X-ray, sagittal CT, sagittal and horizontal MRI preoperatively; (F–J) A–P and lateral X-ray, sagittal CT, sagittal and horizontal MRI 1 day postoperatively; (K–O) A–P and lateral X-ray, sagittal CT, sagittal and horizontal MRI 12 months postoperatively; (P–T) A–P and lateral X-ray, sagittal CT, sagittal and horizontal MRI 24 months postoperatively. CSA expanded after OLIF–AF and expanded further during follow-up. Derotation and partial reduction of slippage were accomplished and stayed relatively stable. DH slightly deceased but CS did not occur, no fixation loosening was observed and fusion was accomplished.

No cerebrospinal fluid leakage, ureteral injury, major vascular injury, endplate injury or nerve damage was observed. No screw misplacement, adjacent segmental disease, fixation failure or operation related infection observed. But 8 patients reported pain in the left thigh and all totally relieved within 3 months. CS occurred in 14 cases but all were mild (Table 5, Fig. 4).

**Table 5:** Complications.

Cerebrospinal fluid leakage	0
Ureteral injury	0
Major vascular injury	0
Nerve damage	0
Screw misplacement	0
Operation related infection	0
Left thigh pain	8
Right thigh pain	0
Cage subsidence	14
Adjacent segment disease	0

## 4. Discussion

SLSS is typically caused by degenerative changes and progresses through various stages. It begins with intervertebral disc degeneration, leading to a loss of DH and instability. Subsequently, there is osteophyte, calcification of posterior longitudinal ligament, thickening and even calcification of ligamentum flavum, resulting in further stabilization.^[17]^ However, this process also leads to narrowing of the central canal, lateral recess and foramina, which cause symptoms. Patients who do not respond to conservative treatments will require surgical intervention.^[4]^

Surgical treatment for decompression can be classified into direct and indirect decompression. Direct decompression like TLIF and PLIF were considered “standard procedures,” but they disturb posterior structures, which is against the opinion of reducing trauma. As for indirect decompression, there was controversy. Lang et al suggested pure indirect decompression may be insufficient for SLSS and should be combined with direct decompression.^[18]^ Oliveira et al also recommended in cases of central canal stenosis, direct decompression should be performed rather than indirect decompression.^[19]^ However, Liu et al and Shimizu et al researches indicated OLIF indirect decompression could effectively improve symptoms of patients with SLSS, and demonstrated OLIF could achieve similar satisfactory therapeutic outcomes when compared to TLIF and PLIF.^[10,11]^ And other indirect decompression methods like ALIF, LLIF, XLIF were also capable of treating SLSS.^[8,20–22]^ Our results indicated OLIF–AF can effectively achieving and maintaining satisfactory pain and functional improvement.

The principle of direct decompression involves the removal of facet joints, lamina, ligamentum flavum, and spinous process to open up spinal canal.^[9]^ OLIF is one of the representative surgical techniques for indirect decompression. Its principle involves removal of degenerated intervertebral discs and placement of intervertebral fusion devices to restore DH and FA, resulting in tension of posterior longitudinal ligament and ligamentum flavum. Partial reduction of the spondylolisthesis can be achieved. We also thinned the posterior longitudinal ligament, especially near the upper and lower endplates, to aid in its restoration. These procedures aim to achieve indirect decompression by relieving central canal, foraminal, and lateral recess stenosis without disrupting spinal canal. FA increased about 41.5% after the surgery and 35.9% at the last follow-up, CSA increased 98.3% right after the surgery and almost 124.5% at 2 years follow-up. During follow-up process we observed ligamentum flavum remodeling and further enlargement of the central canal, which is similar to research conducted by Liu et al.^[11]^ LF was thinned 16.9% after the surgery and it reached 20.7% at 12 months postoperatively. Then it stayed relatively stable. This showed ligamentum flavum remodeling occurred mostly in the 1st postoperative year. This phenomenon may be attributed to the instantly increased stretch on the ligamentum flavum after DH restoration, as well as long term decreased tension due to fixation and fusion. Vertebral rotation was suggested to have a correlation with both facet joint angle and lumbar degenerative conditions.^[23]^ Due to preoperative position placement of patient, trail fitting and fixation, vertebral derotation was achieved as DFA decreased 65.7%, which we think can also contribute to spinal canal expansion and nerve root tension reduction. DFA stayed relatively stable at each postoperative follow-up, showed AF can provide satisfying stability to the spine.

We believe that indirect decompression is suitable for patients with “soft” stenosis caused by conditions such as disc herniation, lumbar instability, spondylolisthesis, and ligamentum flavum hypertrophy. However, if patients have “hard” stenosis caused by conditions such as preoperative spontaneous fusion of facet joints, osseous spinal canal or foraminal narrowing, ligamentum flavum ossification, intervertebral disc calcification, or posterior longitudinal ligament ossification, indirect decompression may be ineffective, and direct decompression becomes necessary.

There are different opinions among scholars regarding the placement of cages. Several studies suggested that cage should be placed anterior-third of the interverbal space to effectively restore the lordotic angle of surgical segment.^[24]^ Generally, we placed the cage in the middle-third of the intervertebral space. However, we believe that for patients with ligamentum flavum hypertrophy, cage position should be as posterior as possible, while ensuring avoidance of major blood vessels, ureters, spinal canal and counter-lateral nerve roots. This facilitates more effective thinning of LF, enlarges CSA, and achieves decompression. When encountering SLSS with poor lumbar lordosis and concomitant hypertrophy of ligamentum flavum, in order to find an appropriate cage placement position to keep balance between restoring lumbar lordosis and thinning the ligamentum flavum, further research is still needed.

Due to the use of the AF, which requires only a single incision to complete the entire OLIF–AF procedure, as opposed to PSF and other posterior approaches, we were able to effectively reduce surgical time from 91.9 to 61.1 minutes and reduce blood loss from 76.7 to 20.9 mL.^[10,11]^ This approach has the potential to decrease the risk of infection and anesthesia-related complications. Additionally, SLSS is often associated with degenerative changes, which can result in variations of posterior anatomical structures. Former research demonstrated a misplacement rate of 41% of pedicle screws, and the possibility of nerve-related complications associated with screw placement.^[25]^ In contrast, AF avoids such difficulties.

This study has the following limitations: it is a single-center retrospective analysis with a relatively small sample size; there was no paired comparison with other surgical methods or fixation techniques; the focus was solely on the L4–5 segment. Future studies should include multi-center designs with larger sample sizes, encompassing other segments and longer follow-up durations. These studies should compare the effectiveness and safety of different surgical techniques and fixation methods for the treatment of SLSS, providing higher-level evidence.

## 5. Conclusions

OLIF–AF can effectively restore DH, FA, and CSA, reducing lumbar rotation, thinning the hypertrophic ligamentum flavum, significantly improving patients’ pain and function, and maintaining efficacy. Additionally, it has few postoperative complications, indicating that OLIF–AF may be a safe and effective treatment for SLSS.

## Author contributions

**Conceptualization:** Jiancheng Zeng.

**Data curation:** Xiandi Wang, Long Zhao, Xingxiao Pu, Liyu Ran.

**Formal analysis:** Xingrui Peng, Xiandi Wang, Long Zhao, Xingxiao Pu, Xiao Hu.

**Funding acquisition:** Jiancheng Zeng.

**Investigation:** Xingrui Peng, Tianhang Xie, Xiandi Wang, Long Zhao, Liyu Ran.

**Methodology:** Xiandi Wang.

**Project administration:** Jiancheng Zeng.

**Resources:** Jiancheng Zeng.

**Supervision:** Jiancheng Zeng.

**Software:** Xiandi Wang, Xingxiao Pu, Xiao Hu, Xu Han.

**Validation:** Tianhang Xie, Xiandi Wang, Xingxiao Pu.

**Visualization:** Xingrui Peng, Tianhang Xie, Xiandi Wang, Xingxiao Pu, Xiao Hu, Xu Han.

**Writing – original draft:** Xingrui Peng, Tianhang Xie.

**Writing – review & editing:** Xingrui Peng, Tianhang Xie, Jiancheng Zeng.
